# 

**Published:** 1899-09

**Authors:** 


### CONTENTS.

### COMMUNICATIONS.

Pathology and Classification of Pulpless Teeth, Without Regard to Abscessed Conditions .......... 397
The Therapeutics of Inflammation.......... 405
Dental Education.......... 409
The Human Face and Jaws as a Danger Signal of Systemic Defect or Disorder.......... 410

### PROCEEDINGS.

National Association of Dental Faculties.......... 412

### SELECTIONS.

In the Far East.......... 408
Experiments with Nirvanin .......... 427
Anthropometry in Children .......... 429
The Influence of Speech Disturbances on Psychical Development ... 430
Bacilli in the Air and Infection.......... 431
The Tongue in Influenza.......... 432
Dental Dispensaries.......... 433
Herr Metzger .......... 433
A Decalogue of Hygienic Maxims.......... 434
A Very Simple Procedure to Remove Moles.......... 434
Buddha's Tooth.......... 435
The New Anesthetics. .......... 435
To Abort a Cold.......... 436
To Keep Needles and Cutting Instruments Bright.......... 436
Habit a Factor to be Considered in the Treatment of Regulation Cases .......... 437
A Movement to Perfect an Academy of Dental Science in San Francisco.......... 437
Mr. William Mitchell, M.B., C.M. Edin., on the Use of Formalin in the Treatment and Removal of Inoperable Malignant Growth.... 438
Dr. Nelson J. Henry Introduced in the Legislature a Bill providing for the Organization of a State School of Public Health.......... 438
The Teeth of Recruits.......... 439
Never Sleep .......... 439
A Certain Variety of Cold for which Gelsemium is as near Specific as any Drug can be.......... 439
Carbolic Acid Dangerous Every Way.......... 440
When We Were Young, Tom.......... 440

### EDITORIAL.

National Dental Association.......... 441
The 13th International Medical Congress .......... 442
College Opening.......... 442
Withdrawal .......... 443

### OBITUARY.

Dr. Charles S. Jones.......... 444

